# Draft Genome Sequences of Bacillus glennii V44-8, Bacillus saganii V47-23a, *Bacillus* sp. Strain V59.32b, *Bacillus* sp. Strain MER_TA_151, and *Paenibacillus* sp. Strain MER_111, Isolated from Cleanrooms Where the Viking and Mars Exploration Rover Spacecraft Were Assembled

**DOI:** 10.1128/MRA.00354-20

**Published:** 2020-06-25

**Authors:** Elinne Becket, Keneshia O. Johnson, Camille J. Burke, Jasmin J. Clark, Marcus J. S. Cohen, David A. Coil, Courtney A. Eggleston, Tyesha L. Farmer, Tiffany R. Farr, Sophia M. Hernandez, Jeff P. Jaureguy, Guillaume Jospin, Afshin Khan, Michael D. Lee, Lauren N. McKee, Erin M. O’Brien, Betsy A. Read, Roxane Saisho, Arman Seuylemezian, Sergio S. Serrato-Arroyo, Dylan Steinecke, Parag Vaishampayan

**Affiliations:** a College of Science, Technology, Engineering, and Mathematics, California State University San Marcos, San Marcos, California, USA; b College of Engineering, Technology, and Physical Sciences, Alabama Agricultural and Mechanical University, Normal, Alabama, USA; c Genome Center, University of California, Davis, Davis, California, USA; d College of Agricultural, Life, and Natural Sciences, Alabama Agricultural and Mechanical University, Normal, Alabama, USA; e Biotechnology and Planetary Protection Group, Jet Propulsion Laboratory, California Institute of Technology, Pasadena, California, USA; f Exobiology Branch, NASA Ames Research Center, Mountain View, California, USA; g Blue Marble Space Institute of Science, Seattle, Washington, USA; Loyola University Chicago

## Abstract

We report the draft genome sequences of Bacillus glennii V44-8, Bacillus saganii V47-23a, and *Bacillus* sp. strain V59.32b, isolated from the Viking spacecraft assembly cleanroom, and *Bacillus* sp. strain MER_TA_151 and *Paenibacillus* sp. strain MER_111, isolated from the Mars Exploration Rover (MER) assembly cleanroom.

## ANNOUNCEMENT

Three strains used in this study, Bacillus glennii V44-8, Bacillus saganii V47-23a, and *Bacillus* sp. strain V59.32b, were isolated from the vehicle assembly building (VAB) at Cape Canaveral, Florida, where the Viking spacecraft were assembled (1). Teflon ribbons were left out for 7 days to collect airborne microorganisms and then exposed to a total of 6 different heat treatments at 3 different time cycles (2). The other 2 isolates, *Bacillus* sp. strain MER_TA_151 and *Paenibacillus* sp. strain MER_111, were isolated from the Mars Exploration Rover (MER) cleanroom.

All 5 strains were cultured in tryptic soy agar (TSA) medium at 32°C for 48 h, and the DNA was extracted using an automated DNA extraction instrument (Maxwell 16, Promega, USA). An Illumina TruSeq DNA PCR-free library preparation kit (350-bp insert size) was used following the manufacturer’s instructions, and paired-end Illumina sequencing was performed on the HiSeq 2500 platform at Psomagen (Rockville, MD, USA). The raw reads were processed with CLC Genomics Workbench v10.1.1, using the default parameters for performing filtering and trimming of adapters and ambiguous nucleotides. The assembly k-mer size was optimized based on the *N*_50_ scores. The quality of the assembled genomes was assessed using QUAST v4.0 (3). The genome statistics were analyzed using Bioinformatic Tools v1.4.71 (4), and the estimated completeness and contamination were evaluated using CheckM v1.1.2 (5). The genomes were subsequently annotated using the NCBI PGAP pipeline v4.6 (V44-8, V47-23a, and V59.32b) and v4.9 (MER_TA_151 and MER_111) (6). See Table 1 for information on the assemblies and for the annotation summaries of the five strains.

**TABLE 1:** Sequencing and assembly metrics and NCBI PGAP annotation data for bacterial strains

Statistic	Data for strain:				
	V44-8	V47-23a	V59.32b	MER_TA_151	MER_111
Project accession no.	QVTD00000000.1	QVTE00000000.1	QVTC00000000	VYKL00000000	VYKK00000000
No. of raw read pairs	6,707,121	8,523,831	6,008,123	6,558,078	5,667,551
Assembly size (bp)	4,469,041	4,356,520	3,830,155	5,743,622	4,305,989
No. of contigs >1,000 bp	27	84	123	85	45
*N*_50 (bp)_	242,250	81,582	57,552	146,989	224,736
*L*_50_	4	15	23	11	6
GC content (%)	42.26	40.58	41.71	37.86	56.99
Estimated completeness (%)	98.91	98.09	98.36	99.33	99.07
Estimated contamination (%)	0.96	1.81	1.73	6.62	1.18
No. of identified genes (total)	4,358	4,109	3,803	5,427	3,905
No. of identified CDSs^*a*^ (total)	4,286	4,028	3,722	5,326	3,828
No. of complete rRNAs (5S, 16S, 23S)	4, 1, 1	2, 1, 0	0, 1, 0	1, 0, 0	2, 1, 0
No. of predicted tRNAs	60	71	73	83	69
No. of predicted ncRNAs^*b*^	6	6	6	12	4

a CDSs, coding DNA sequences.

b ncRNAs, noncoding RNAs.

The taxonomic assignments of *B. glennii* and *B. saganii* were determined based on a polyphasic study, including the biochemical, phylogenetic, and phenotypic characteristics (1). GToTree v1.4.11 (7) was used to create a phylogenomic tree with NCBI-designated representative genomes (as accessed on 14 February 2020) of *Bacillus* and *Paenibacillus* based on the concatenated alignments of 119 single-copy core genes specific to the *Firmicutes* phylum (default settings used other than “-H Firmicutes”) (8–14). The genus-level taxonomies of the *Paenibacillus* isolate, *Bacillus* sp. strain V59.32b, and *Bacillus* sp. strain MER_TA_151 were determined by their positions in the phylogenetic tree (as shown in https://doi.org/10.6084/m9.figshare.12245441). We were unable to assign species-level taxonomy to these isolates due to the known discrepancies between phylogeny and taxonomy in these genera.

### Data availability.

The whole-genome shotgun sequencing projects were deposited in GenBank and the raw sequencing reads in the NCBI Sequence Read Archive under the accession numbers QVTD00000000.1 and SRR11096019 (*Bacillus glennii* V44-8), QVTE00000000.1 and SRR11096037 (*Bacillus saganii* V47-23a), QVTC00000000 and SRR11097317 (*Bacillus* sp. strain V59.32b), VYKL00000000 and SRR11096322 (*Bacillus* sp. strain MER_TA_151), and VYKK00000000 and SRR11097201 (*Paenibacillus* sp. strain MER_111), respectively.
